# Investigating the practical viability of walk-sharing in improving pedestrian safety

**DOI:** 10.1007/s43762-021-00020-z

**Published:** 2021-09-06

**Authors:** Debjit Bhowmick, Stephan Winter, Mark Stevenson, Peter Vortisch

**Affiliations:** 1 Department of Infrastructure Engineering, The University of Melbourne, Melbourne, Australia; 2 Transport, Health and Urban Design Research Lab, The University of Melbourne, Melbourne, Australia; 3 Institute for Transport Studies, Karlsruhe Institute of Technology (KIT), Karlsruhe, Germany

**Keywords:** Walking, Walk-sharing, Agent-based modelling, Pedestrian safety, Fear of crime, Urban planning

## Abstract

Walk-sharing is a cost-effective and proactive approach that promises to improve pedestrian safety and has been shown to be technically (theoretically) viable. Yet, the practical viability of walk-sharing is largely dependent on community acceptance, which has not, until now, been explored. Gaining useful insights on the community’s spatio-temporal and social preferences in regard to walk-sharing will ensure the establishment of practical viability of walk-sharing in a real-world urban scenario. We aim to derive practical viability using defined performance metrics (waiting time, detour distance, walk-alone distance and matching rate) and by investigating the effectiveness of walk-sharing in terms of its major objective of improving pedestrian safety and safety perception. We make use of the results from a web-based survey on the public perception on our proposed walk-sharing scheme. Findings are fed into an existing agent-based walk-sharing model to investigate the performance of walk-sharing and deduce its practical viability in urban scenarios.

## Introduction

### Background

*Walking* is the most common mode of travel given its high levels of accessibility, especially for short trips (Hong and Chen 2014; Zielstra and Hochmair 2012). Apart from being an independent mode of transport, walking also serves as the most common mode of access to public transport (Bassett et al. 2008). Multiple studies have revealed that the physical activity generated by walking improves people’s physical health (Giles-Corti et al. 2016; Warburton et al. 2006). Others have shown the positive impacts of walking on mental health (Besser and Dannenberg 2005; Donnelly et al. 2000; Lee and Buchner 2008; Johansson et al. 2011). Among young-adult Australians, walking for transport alone accounts for nearly half of their daily physical activity (Garrard 2017). Walking contributes beyond physical and mental health improvements. It has community benefits as well. As destination-focused walking increases, the use of automobiles goes down for trips that would be otherwise convenient for walking. This consequently reduces traffic congestion on roads, energy consumption, air and noise pollution by motorised vehicles, and other related expenses (Pucher and Buehler 2010; Stevenson et al. 2016). All of this can lead to more liveable communities (Zhang and Mu 2019).

However, challenging walking environments discourage people from walking. Inconvenience acts as the major challenge for outdoor walking. For example, trips that are too long (for non-leisure walking trips), or walking in adverse weather conditions will not appear attractive to most. But, even under convenient circumstances, certain environments induce the sense of fear in pedestrians (Colls and Evans 2014; Ferrer and Ruiz 2018; Ferrer et al. 2015; Nasar 1990; Powdthavee 2005; Ross 2000; Sakip et al. 2012). This stems from the physical vulnerability of pedestrians who travel with lower speeds and are in an unprotected state as compared to other travel modes (Wegman et al. 2008). Existing studies have shown that personal safety is an area of concern among pedestrians in the urban context (Doeksen 1997; Halat et al. 2015; Warr and Ellison 2000). Fear of crime has been cited as the most important barrier for which walking becomes unattractive during critical hours of the day (e. g. after-dark hours), even though walking might be convenient otherwise (Ferrer and Ruiz 2018). It prevents pedestrians, mostly women and the elderly, from walking alone in public places after dark (Franc and Sucic 2014; Garrard 2017). Being fearful while walking has detrimental consequences on both the individual and the community (De Silva et al. 2017; Nasar 1983).

Human mobility decisions (choice of route and travel mode) are influenced by fear under critical circumstances (Rodríguez et al. 2015). When feeling vulnerable, pedestrians often take detours to avoid fearful places (Borst et al. 2009; Michael et al. 2006; Park et al. 2011; Pun-Cheng and So 2019). Fear of crime leads pedestrians to abandon or minimise walking, and switching to costlier alternative travel modes (Ferrer and Ruiz 2018; Hong and Chen 2014). It reduces the attractiveness of walking as a viable and convenient mode of travel. The existence of places associated with fear of crime reduces the walkability of urban spaces, restricts outdoor walking and other related activities (De Silva et al. 2017). This ceases the benefits that are offered by walking (Ross 1993; Taylor 1987). Moreover, it promotes motorised traffic even for short distance trips which negatively impacts the urban liveability.

### Motivation

While traditional approaches aimed at reducing *fear of crime* amongst pedestrians, such as improvements in urban design, or installation of street furniture, have been employed over decades, they are usually costly, never holistic, and take significant time before coming into effect (Day et al. 2007; Fotios and Castleton 2016; Fisher and Nasar 1992; Ferrer et al. 2015; Painter 1994). Other, less traditional approaches, such as safe route recommendation systems, or crowdsourced safety ratings of places, have some major drawbacks, such as dependency on historical crime records or proxy social media data which is sparse (Fu et al. 2014; Kim et al. 2014; Utamima and Djunaidy 2017). With the advancement of technology, ubiquitous computing and smartphone sensors, we look at tackling the challenge of reducing fear of crime with a novel approach.

Existing literature suggests that the absence of other people is the major reason for pedestrians feeling fearful while walking through urban spaces at critical times of the day, even when elements of the infrastructure are conducive for walking (such as sufficient street lighting) (Ferrer and Ruiz 2018). Pedestrians feel safer when they walk with a companion as compared to walking alone in environments perceived as unsafe (Clifton and Livi 2005). The presence of just one other pedestrian nearby boosts natural vigilance which is not perceived as favourable by a potential offender (Iglesias et al. 2013; Painter 1994). The presence of a walking companion enhances both security and sense of security, and thereby reduces perceived risk and the fear of victimisation significantly (Cohen and Felson 1979). Hence, a person could opt for walking as they feel safer when they share their walking trip with a trustworthy walking companion instead of walking alone. This may include a friend, a family member (walking to the nearest supermarket from home) or a colleague (walking from the workplace to the nearest train station). But, a pedestrian is not guaranteed a walking companion under all critical circumstances. To overcome this challenge, in an earlier study, we proposed *walk-sharing*, a cost-effective and proactive approach to improve pedestrian safety (Bhowmick et al. 2021). Walk-sharing is a hypothetical buddy-service, where potential pedestrians get matched to each other and would share their walking trip. Walk-sharing exploits spatio-temporal overlap of people’s trip details to match people with each other, while trying to minimise related costs. This would ensure a walking companion, albeit unknown, and thus encourage people to walk rather than availing alternate modes of transport, especially when walking is viable. In our previous study, we had introduced the fundamental concepts of walk-sharing, produced theoretical insights, developed an agent-based simulation model, and tested the technical viability of walk-sharing in a real-world data-driven scenario. We had shown that walk-sharing was technically viable at critical times of the day as it was delivering acceptable levels of performance metrics (also discussed in Section 2.3) along with significant safety improvement for pedestrians.

But, we were ill-informed about the perceptions of the community on walk-sharing. Walk-sharing is intended to improve the safety perception of people, reduce fear of crime, and thereby improve urban liveability. Hence, it is necessary to investigate the perceptions and understand the preferences of the people in the community, who are the potential end-users of this walk-sharing scheme. Gaining useful insights on the community’s spatio-temporal and social preferences can help us go one step ahead and establish the practical viability of walk-sharing in real urban scenarios.

### Research questions

We hypothesise that incorporating stated preferences of the community into the existing walk-sharing model will affirm the fact that walk-sharing can be implemented as a cost-effective tool to improve pedestrian safety. In other words, walk-sharing will be practically viable. Thus we aim to address the following research questions:

- What are the conditions of public acceptability of walk-sharing?
  - What are the thresholds of space-time compromises that people are willing to make to avail walk-sharing?
  - Are there any social preferences that may affect the uptake of walk-sharing?
  - Does the likelihood of taking up walk-sharing vary across demographic groups?
- To what extent can walk-sharing practically improve pedestrian safety in a real-world urban scenario?

### Methodology

To understand the public perception on our proposed walk-sharing scheme, we conducted a web-based survey to gather knowledge about the perceptions of people on walk-sharing. We predict, that through the survey, we would be able to discover interesting elements about the spatio-temporal and social preferences of people, specifically about when, where, why and with whom they would be likely to avail walk-sharing. We plan to state the findings from the survey and consequently use these findings to calibrate our existing agent-based model (with a modified matching algorithm and multi-class agents) with informed choices of spatio-temporal and social parameter thresholds and distributions. After running the calibrated model, we compared the current findings with our previous results under the same scenario, and understand the effects of demographics and preferences on the viability of walk-sharing. More importantly, this study will investigate and establish the practical viability of walk-sharing, and thereby, the approach could be used to improve pedestrian safety, given our calibration based on public feedback. We aim to measure improved pedestrian safety in terms of walk-alone distance saved (using objective measure *safety index*) as people feel and are safer when walking in company as compared to walking alone.

### Contributions to knowledge

The first contribution of this paper is knowledge on the community acceptance of walk-sharing. This involved gathering information from the public on their perceptions on walk-sharing if it was in place, such as how likely would they be to avail walk-sharing, or what their spatio-temporal and social preferences would be. Consequently, the analysis of survey responses aimed to gain deeper insights on the distributions of the responses to each perception and preferences, and understand whether these are governed by the socio-demographics of the respondents.

The second contribution of this paper is establishment of the practical viability of walk-sharing in a real-world data-driven urban scenario by calibrating an existing agent-based model with parameter thresholds and distributions derived from the survey. This way the practical viability of walk-sharing could be objectively measured using relevant performance metrics viz. waiting time, detour length, walk-alone distance, matching rate, and safety index.

## Literature review

### Fear of crime in pedestrians

*Fear of crime* or criminal victimisation is a major challenge in the urban context. Policymakers and researchers have concerned themselves heavily over the subject of fear of crime among pedestrians in the last decade (Roberts 2014). One prominent study had concluded that *“such fear continues to impinge upon the well-being of a proportion of the population”* (Gilchrist et al. 1998). Since the 1960s, researchers have examined the safety perception of individuals walking in their local area during night time (Roberts 2014). In Chicago, Illinois, fear of crime has been found to significantly reduce the likelihood of outdoor walking (Ross 1993). In Australia, 36% people stated that do feel unsafe while walking alone at night (Jericho 2017). A survey conducted in 2015 by Plan International across four countries stated that a majority of people agreed to the fact that women are not safe in public spaces after dark (Plan International Australia and Our Watch survey 2016). Around 50% women and 20% men in the U.S. said they are afraid to walk alone at night, even in their own neighborhoods (Badger 2014). Results from surveys conducted by Gallup and the National Opinion Research Center spanning over four decades have revealed that over a third of the respondents have fears of criminal victimisation when walking alone at night (Roberts 2014).

### Existing approaches for pedestrian safety perception improvement

Fear of crime in pedestrians has been proven to be detrimental for the society. Hence, researchers have been looking at ways to reduce fear of criminal victimisation while walking and improve safety perception of pedestrians in urban spaces. In this context, several studies have made significant contributions as they have proposed different solutions to tackle this problem. We have classified these methods into two broad classes. The first class of studies have investigated specific built environment attributes that influence crime, fear of crime and thus safety perception (Adel et al. 2016). These attributes can be divided into macro attributes (residential density, land use-mix and route connectivity) and micro (safety, pedestrian infrastructure and aesthetics) (Zandieh et al. 2016). Drawing on the results of these studies, others have proposed efficient methods of installation or removal of these attributes (Day et al. 2007; Fotios and Castleton 2016; Fisher and Nasar 1992; Ferrer et al. 2015; Painter 1994). The second class of studies propose location-based low-cost IT approaches, such as developing novel *safe route* recommendation services that avoid possible unsafe locations. They use historical crime data or semantically analyse proxy georeferenced social media data in this regard (Fu et al. 2014; Kim et al. 2014; Utamima and Djunaidy 2017).

While these traditional approaches of reducing fear of crime amongst pedestrians are more established, they still suffer from a few major drawbacks. For the first class of studies, the macro attributes (alleyways, deserted areas, vacant land parcels, empty green-spaces) take significantly large time periods to be modified, even modification of the micro attributes (street lighting, alcoves, tall dense shrubs, blind spots, graffiti) involve significant time and money. Hence, it is challenging for the local authorities to holistically mitigate all such factors that discourage people from walking. For the second class of studies that rely on location-based data and IT approaches, the drawbacks are manifold. First, crime is an outlier, which means it is rare and an unusual event in space-time. On the contrary, fear of crime is not restricted to space and time and is more widespread than actual crime (Brown et al. 2000; Oc and Tiesdell 1997). Fear of crime has a much greater influence on the safety perception of pedestrians than crime itself (Matei et al. 2001). Second, there are many such locations where people feel vulnerable but such locations have no historical crime records. Given these methods are data-dependent, they are ‘reactive’ and hence suffer in the absence of relevant data. Third, crime data and proxy social media data suffer from their drawbacks when trying to represent fear of crime. They are not always correlated with fear of crime and run the risk of under-representing or over-representing fearful places. Fourth, navigation decisions based on historical crime records and tweet sentiments indirectly create ghettos by isolating such areas even further. Systems with such approaches have the potential to profile racial and socio-economic groups (Wood et al. 2021). This is detrimental for the overall walkability of the area, is less inclusive in nature, and hence, not desirable.

### Previous study on walk-sharing

In our previous study (Bhowmick et al. 2021), we had introduced *walk-sharing*. Walk-sharing matches potential pedestrians in a pairwise manner, so that they can walk with a companion, instead of walking alone. This way they avoid walking alone and thereby overcome their fear of criminal victimisation that arises out of seemingly unsafe walking environments. The presence of a walking companion not only improves safety perception, but, by increasing natural vigilance, it also reduces the likelihood of victimisation. This is crucial, especially in outdoor environments that are sparsely populated, such as deserted streets after dark. In the presence of sufficient ambient pedestrian population, people may not feel the need for availing walk-sharing. But in critical circumstances, presence of pedestrians along the entire route cannot be guaranteed. Walk-sharing attempts to utilise spatio-temporal overlap in journey times and routes between any two potential pedestrians. This way they can walk together while the system tries to optimise related costs such as waiting time, detour distance. Walk-sharing is a cost-effective intervention that attempts to reduce fear of crime among pedestrians. It does not require municipal authorities to make design overhauls or revamp street furniture and allocate hefty budgets. It is also a *proactive* method, meaning that contrary to existing reactive and data-dependent methods, it is scalable (can be extended to cover larger areas) and transferable (can be implemented anytime anywhere). It is also *more inclusive* in nature, as it does not create ghettos by recommending users to bypass certain locations, and thereby does not participate in socio-economic and racial profiling unlike now-defunct applications such as *SketchFactor* and *Ghetto-Tracker* (Greenfield 2013; Dewey 2014; Wood et al. 2021). In principle, walk-sharing has the potential to reduce both crime and fear of crime.

**Fig. 1:**
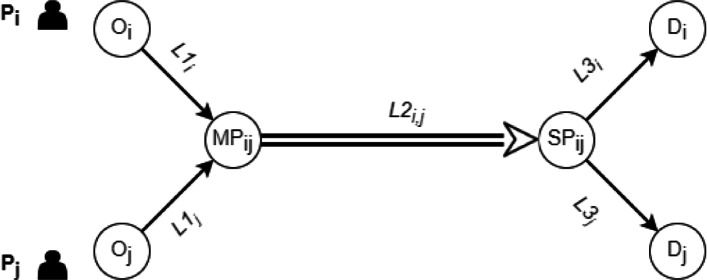
Schematic framework of walk-sharing

We illustrate the schematic framework of walk-sharing in Fig. 1 proposed by Bhowmick et al. (2021). *P*_*i*_ and *P*_*j*_ are two people willing to participate in walk-sharing. After a successful matchmaking process (matched to each other), they start from their respective origins (*O*_*i*_ and *O*_*j*_) and walk to their designated meeting point (*MP*_*ij*_). After meeting, they start walking together, and thus share their walk till their designated separation point (*SP*_*ij*_). From here, they walk alone towards their respective destinations (*D*_*i*_ and *D*_*j*_). The scope of this study is limited to pairwise matching only. This means in any single walk-sharing event, the number of participants is fixed at two.

We had also defined the costs of walk-sharing, aspects that act as inhibitions from participating in walk-sharing. These costs also act as performance measures, meaning metrics that assist in the objective measurement of the performance of walk-sharing. These are defined as follows, as proposed by Bhowmick et al. (2021).

- **Waiting time** - The time difference between a pedestrian becoming active in the system (at the time in which they want to start their walking journey), and the time at which the system returns a match.
- **Walk-alone distance** - The distance that a pedestrian has to walk alone while participating in walk-sharing. It is the sum of two walking distances, first between the origin and the meeting point and the second, between the separation point and the destination.
- **Detour length** - The difference between the actual route length (while participating in walk-sharing) and the shortest route length (if not participating in walk-sharing and walking alone directly to the destination).
- **Matching rate** - The percentage of the population (people who want to avail walk-sharing and are hence active on the system) that is matched with a companion. Matching rate is not a cost, but a critical performance metric for walk-sharing.

**Fig. 2:**
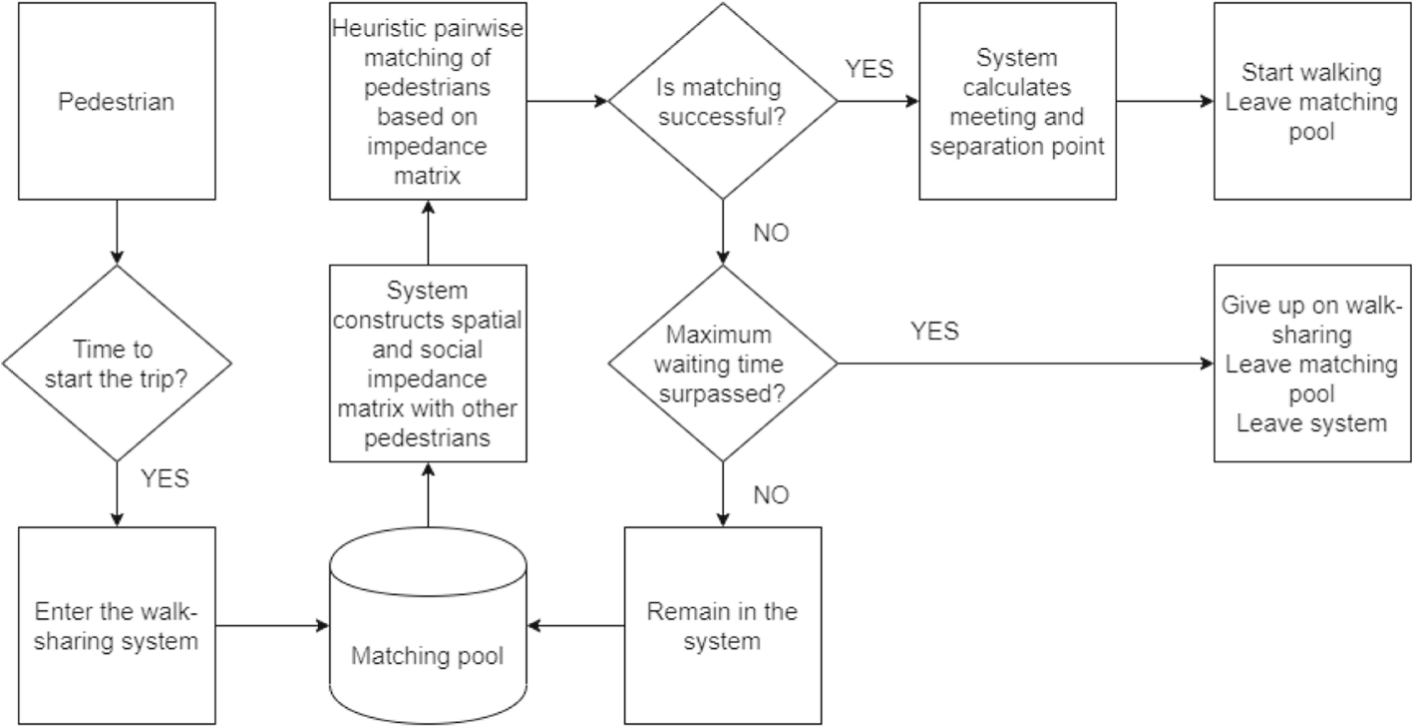
Conceptual model of the proposed walk-sharing system

The conceptual model of walk-sharing is illustrated in Fig. 2. We had realised this conceptual model by converting it into an agent-based model. This was done for the purpose of being objectively able to measure the performance and effectiveness of walk-sharing. To construct the walk-sharing model, we used an agent-based simulation platform called GAMA (GIS Agent-based Modeling Architecture) (Lao 2019; Iskandar et al. 2020). The model simulates pedestrians moving along the pedestrian road network, starting from an origin and reaching a destination. Pedestrian agents are activated when the system time corresponds with their starting time, and is consequently included in the matching pool where they wait to be paired up with another pedestrian agent. These agents are matched as per the spatio-temporal and social preferences set in the model. The matching algorithm uses a minimum distance to buddy heuristic approach as shown in Algorithm 1. Pedestrians are matched pairwise and exclusively, meaning two pedestrians at a time and multiple matches are not returned. After matchmaking, the matched agents are taken out of the matching pool. At every time step, the matching pool is refilled with new pedestrian agents who become active. They can wait till they reach their maximum waiting time threshold, and if they remain unmatched still, they give up on walk-sharing, and walk alone to their destination.

First, we ran simulations with synthetic data to establish *proof of concept* (obtaining intuitive results by simulating the walk-sharing model under controlled conditions of parameter values and thereby proving the logical efficacy of the conceptual model). Finally, we used real-world human mobility data from university’s WiFi access records (as described in Section 4.2), and ran simulations showing the times when walk-sharing was theoretically viable in the university scenario. Given that we were not informed about the actual preferences of people, our base model was coarse, and fed with assumed spatio-temporal thresholds, and did not consider social preferences. Hence the obtained results were devoid of calibration based on more realistic thresholds, and therefore the obtained viability was exaggerating (as shown in Section 5). This study aims to investigate community preferences on walk-sharing and obtain relevant thresholds and distributions regarding both spatio-temporal and social preferences of people. These will consequently be fed into the calibrated and more sophisticated walk-sharing model to obtain realistic results on the performance of walk-sharing, thereby establishing its practical viability.

## Community preferences on walk-sharing

This section discusses in detail about a survey that was conducted to obtain useful insights on how people could perceive walk-sharing. While it was established in our previous study that walk-sharing is technically viable in certain real-world scenarios, community preferences in our agent-based model were not accounted for. To obtain information on public feedback and perception about walk-sharing, a survey was deemed necessary. Given that walk-sharing is a novel and hypothetical matching service which is in its conceptual stage and far from realisation, only a stated-preference survey could be conducted. Similar stated-preference (SP) surveys are well-established in the urban and transportation planning domain, given the greater amount of control the researchers have while defining the conditions, and the flexibility of defining new variables (Kroes and Sheldon 1988). SP surveys can help understand a respondent’s evaluation of a product or service, especially in cases where the product or service in question are hypothetical, as it is with walk-sharing.

### Survey instrument

A web-based questionnaire survey was designed for this purpose and was launched on Amazon Mechanical Turk, a commercial survey platform. We confined the recruitment to respondents who, at the time of the survey, resided in urban and suburban locations in Australia, were above 18 years of age, and did not require assistance while walking. Participation in the survey was voluntary. To encourage participation, the respondents were paid 7 Australian Dollars each, an amount that was determined by the existing minimum national wage rate, considering the time required to complete the survey. The survey had ethics approval from the University of Melbourne (Ethics ID: 2057008). The survey was live from June 2020 through October 2020. During that period, it had collected responses from 234 participants. The respondents were briefed about the proposed walk-sharing scheme before starting the survey. The survey collected data related to the attitude of the respondents towards the hypothetical walk-sharing scheme, their spatio-temporal and social preferences. The survey also collected socio-demographic data of the participants to investigate the effect of demographic groups on a participant’s affinity towards walk-sharing.

### Sample characteristics

The demographic characteristics of the 234 respondents have been illustrated in detail in Table 1. The share of each demographic subgroup has been shown in the table. The respondents were predominantly male (69%) and belong to the age bracket of 25-44 years (62%). 76% of them identified English as their used language in household communications and 53% were born in Australia. 67% of the respondents had an educational qualification of Bachelor’s degree or above, more than 60% were working, with uniform representation across income levels. 70% of them had access to a vehicle, and most people used either private motorised or public transport (PT) for commute. When it comes to travelling to the supermarket, around 52% of people use private motorised transport, while 37% of people choose walking as their preferred mode of travel.

**Table 1:** Demographic characteristics of respondents

Characteristic	Categories	Respondents (count)	Respondents (%)
Age	18-24	78	33.3
	25-44	145	62.0
	45-64	11	4.7
Gender	Female	70	29.9
	Male	162	69.2
	Other	2	0.9
Educational qualification	Bachelor’s degree level and above	158	67.5
	Certificate level IV or III or Advanced Diploma and Diploma level	31	13.2
	Year 12 or below	45	19.2
Employment status	Full-time student	53	22.6
	Unemployed	39	16.7
	Work full-time	86	36.8
	Work part-time	56	23.9
Weekly income	0 - 500 AUD	92	39.3
	1001- 2000 AUD	46	19.7
	501 - 1000 AUD	77	32.9
	More than 2000 AUD	19	8.1
Number of vehicles	None	40	17.1
owned	1	81	34.6
	2	77	32.9
	3	17	7.3
	More than 3	19	8.1
Whether has access to	Yes	166	70.9
a vehicle	No	68	29.1
Place of birth	in Australia	126	53.8
	outside Australia	108	46.2
Household language	English	178	76.1
	Any other European language	17	7.3
	Non-European language	39	16.7
Mode of travel to work	Bicycle only	12	5.1
	Private motorised transport (Car, Motorbike) as a driver or a passenger	86	36.8
	Public transport (Tram, Train, Bus)	107	45.7
	Cab	1	0.4
	Walk only	28	12.0
Mode of travel to	Bicycle only	7	3.0
supermarket	Private motorised transport (Car, Motorbike)	121	51.7
	Public transport (Tram, Train, Bus)	15	6.4
	Cab	3	1.3
	Walk only	88	37.6

### Spatio-temporal preferences

**Fig. 3:**
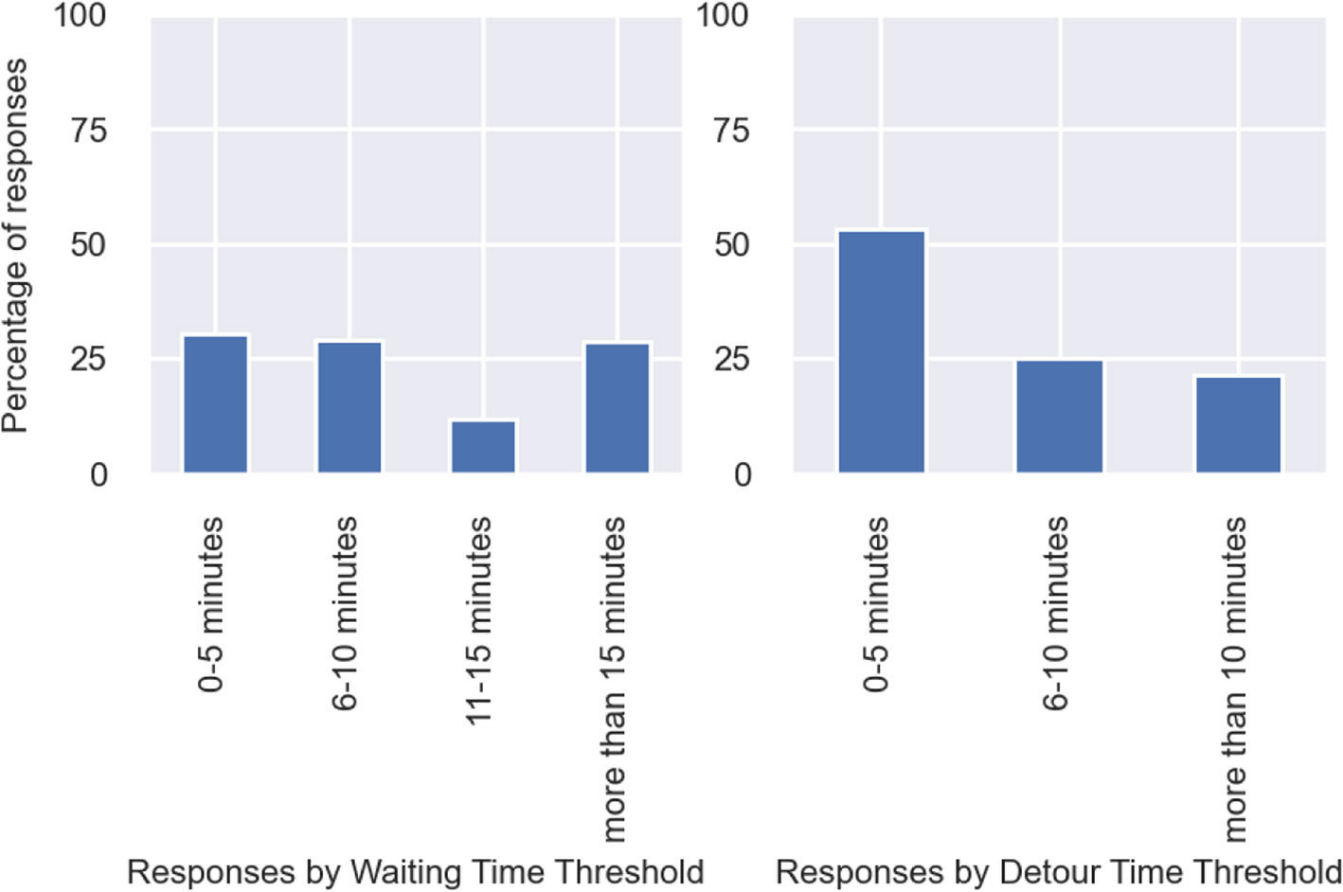
Distribution of responses on temporal preferences

*Waiting time* is the duration for which a person willing to participating in walk-sharing waits before getting matched with a walking companion. We asked people about the maximum time they would prefer to wait to get matched with a walking companion. The results are shown in Fig. 3. It can be observed that except a small fraction of people who say that they are not willing to wait at all, the majority are willing to wait ranging from 1 to more than 15 minutes, which is positive for walk-sharing. It can be seen in Fig. 3 that most of the respondents are either willing to wait up to 5 minutes or 10 minutes, while a significant share of respondents have stated their willingness to wait beyond 15 minutes as well. Interestingly, more people are willing to wait for more than 15 minutes than people who have a maximum waiting time threshold between 11-15 minutes. *Detour time* is a resultant of pedestrians accommodating for their companion’s travel route. Since pedestrians will not walk directly from their origin to their destination, there will be an amount of detour involved. This extra time required to travel to their destination is the detour time. We asked people the maximum detour they are willing to accept when availing walk-sharing. The results are shown in Fig. 3. It can be observed that most people admit to a maximum detour time of 0-5 minutes while some are willing to accept longer detours.

**Fig. 4:**
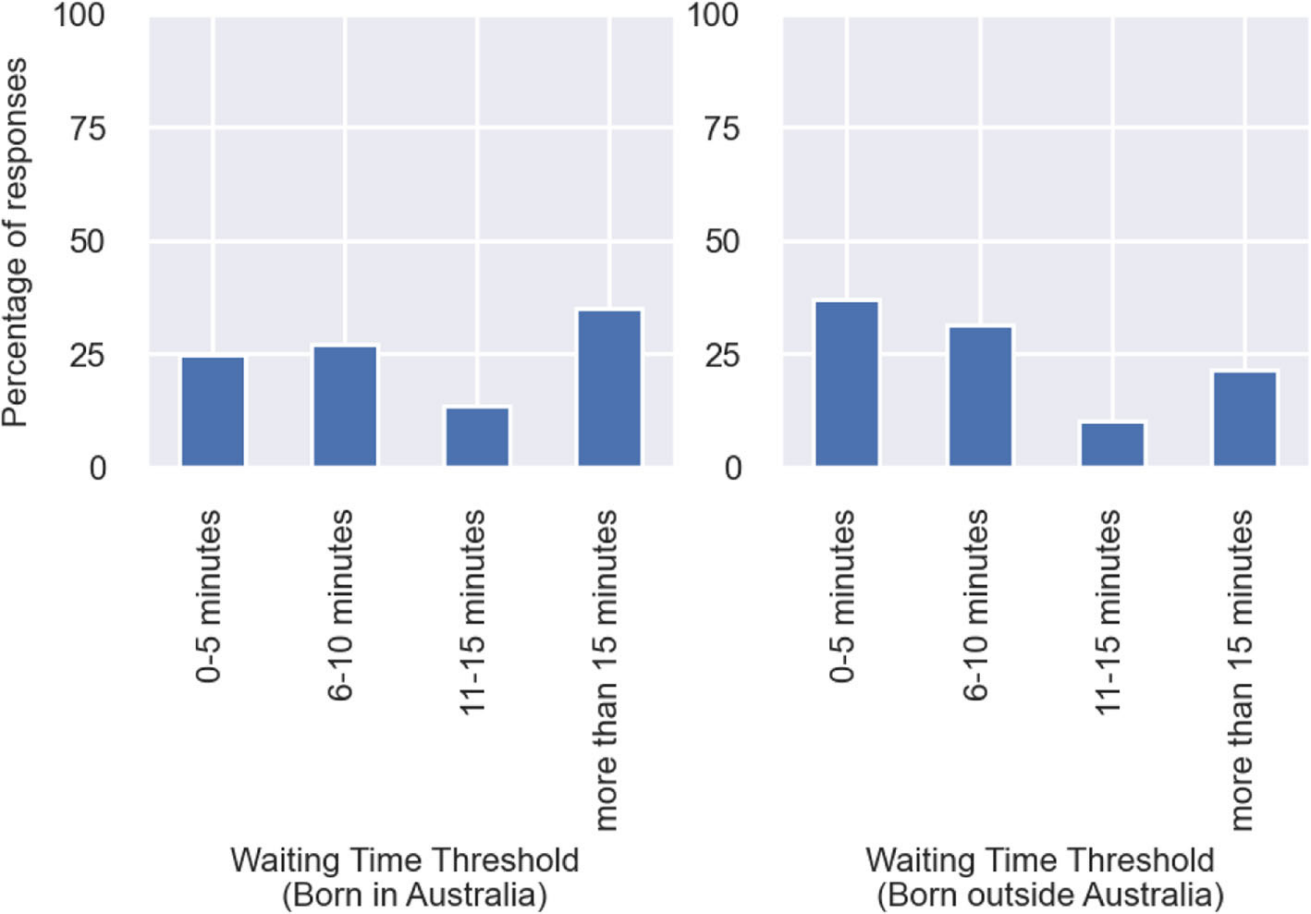
Distribution of responses on maximum waiting time by place of birth

We also wanted to understand whether demographics play a part in governing maximum waiting time and maximum detour time preference stated by the respondents. Since we did not ask for discrete values of maximum waiting time or detour time from the responses, determinate quantitative analysis was not trivial. Hence we visually inspected whether the distribution of maximum waiting time and maximum detour time was different, across demographic subgroups. For waiting time, we found significant differences between the stated waiting time distributions of respondents born in Australia and outside Australia. This can be observed from Fig. 4 where it can be clearly seen that the respondents born in Australia seem to be more flexible in terms of their waiting time thresholds. 35% of those respondents are willing to wait for more than 15 minutes to avail walk-sharing under critical circumstances, while the corresponding figure is 21% for the respondents born outside Australia. We feel this could be due to cultural differences in safety perception among these two demographic groups. But we found no evidence of this sort, especially in the ridesharing domain.

For detour time threshold, we could not find sufficient evidence to explain it as a function of any socio-demographics of the respondents.

**Fig. 5:**
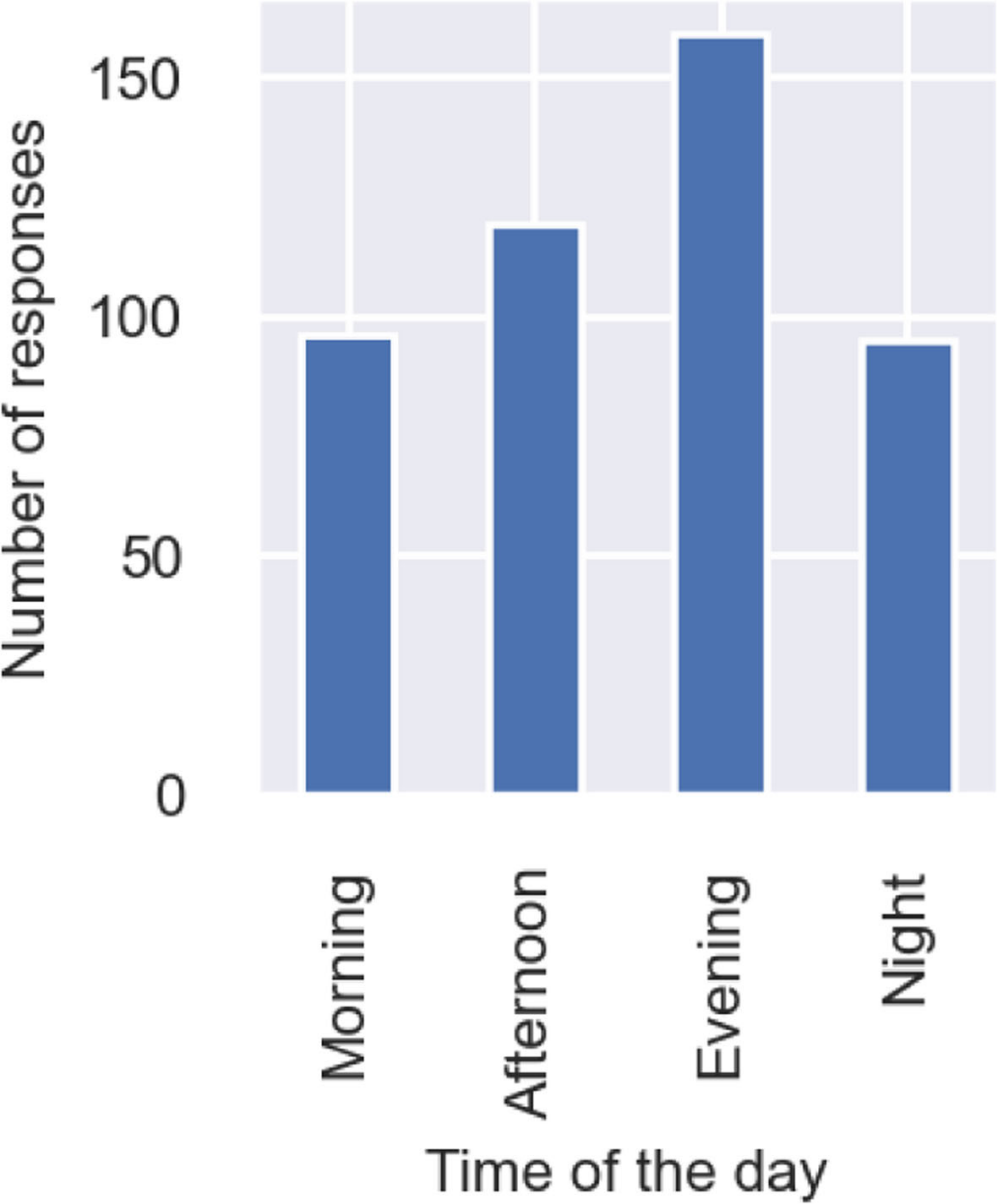
Distribution of responses on preferred time of the day to avail walk-sharing

We also asked people at what times of the day would they prefer to avail walk-sharing. It can be observed from Fig. 5 that many of the respondents have stated that they would prefer to avail walk-sharing in the evening. This could be due to the fact that it gets dark during the evening, and it coincides with a significant amount of pedestrian movement, especially the last leg of people’s journeys back home after a day’s work or study.

### Social preferences

**Fig. 6:**
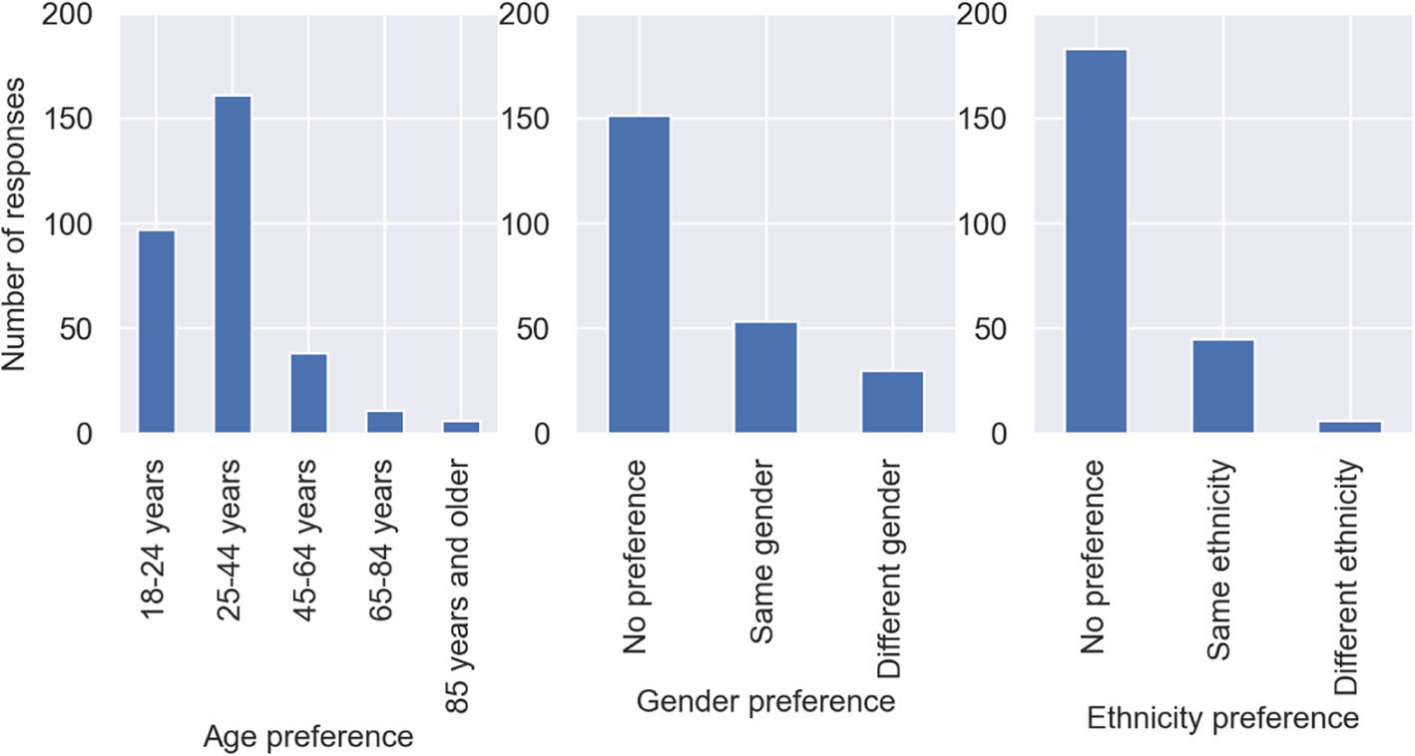
Distribution of responses on social preferences

We expected that there could be social preferences along with spatio-temporal preferences when it comes to walk-sharing. In other words, people may have certain preferences when it comes to the demographics of their companion. We asked the participants what would they prefer when it comes to the age, gender and ethnicity of their walking companion. The distribution of the responses have been illustrated in Fig. 6. It can be observed that the most commonly preferred age groups are 18-24 years and 25-44 years. Given that 85% of respondents belong to these to age groups, it becomes clear why the companion preferences belonging to these two age groups are high. Overall, this shows that people would prefer to have a walking companion who is roughly in the same age group. This could be due to similar mobility attributes such as walking speed and greater chances of socialising during walk-sharing. In terms of gender and ethnicity preferences, majority of the respondents stated that they do not have any preferences, more so for ethnicity.

**Fig. 7:**
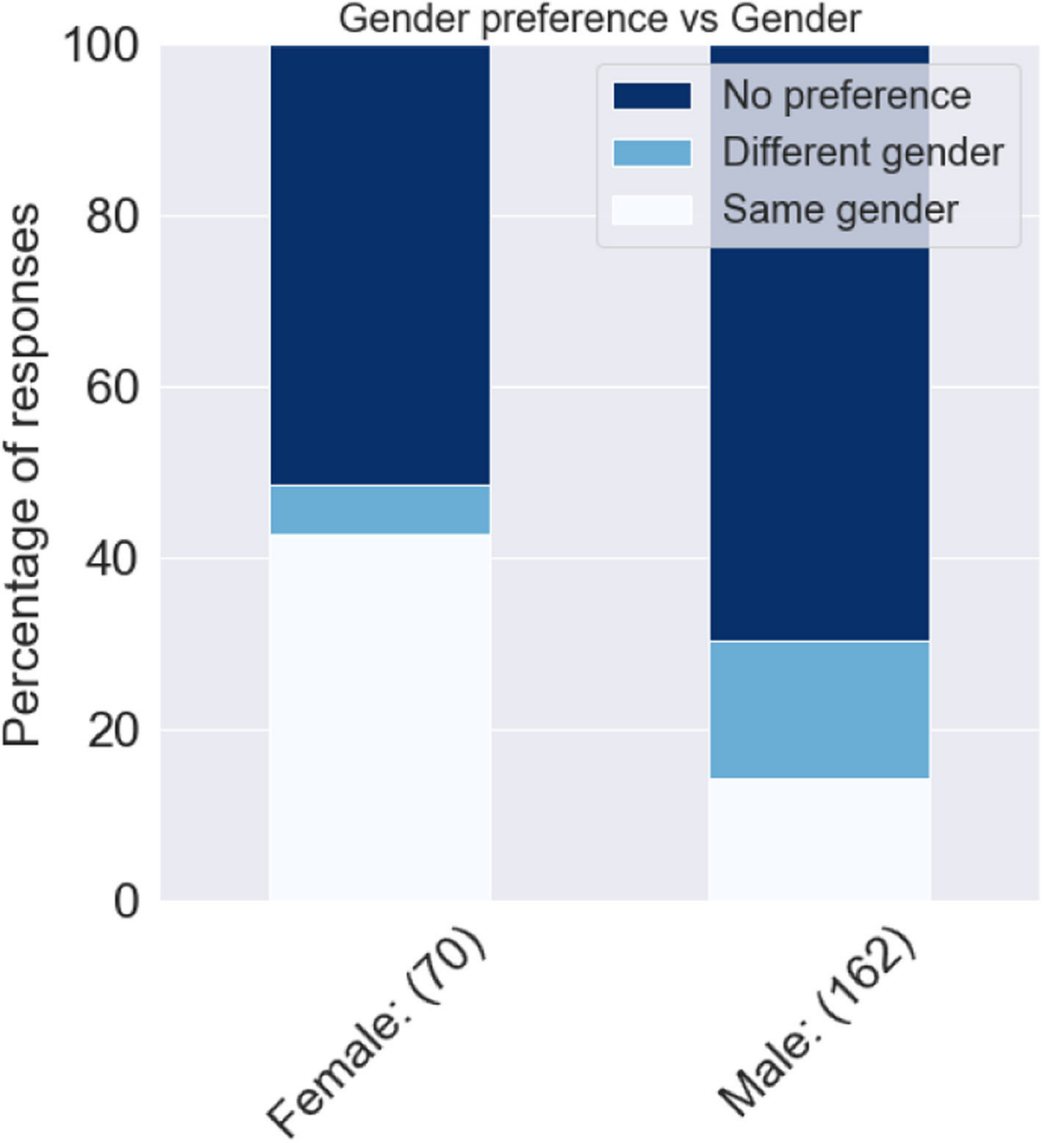
Distribution of responses on gender preference of walking companions

We investigated whether the social preferences of the respondents were influenced by their demographics. After cross-tabulating the three social preferences (age, gender and ethnicity) with all the attribute levels of each demographic variable, we could not find any significant relationship except gender preference, which was found to be significantly varying with the gender of the respondent. It can be observed from Fig. 7 that around 50% of the female respondents stated that they would prefer their walking companion to be of the same gender, which was significantly larger as compared to the 10% of male respondents who said so. In other words, women would be inclined towards walk-sharing more when their assigned walking companion would be another woman. This has been historically observed in ridesharing surveys where women respondents have stated that they felt safer when their co-passenger was another woman, as compared to a man (Meshram et al. 2020). Furthermore, women’s focus of fear in the context of personal safety in outdoor environments is mostly men (Lorenc et al. 2013).

### Likelihood of availing walk-sharing

**Fig. 8:**
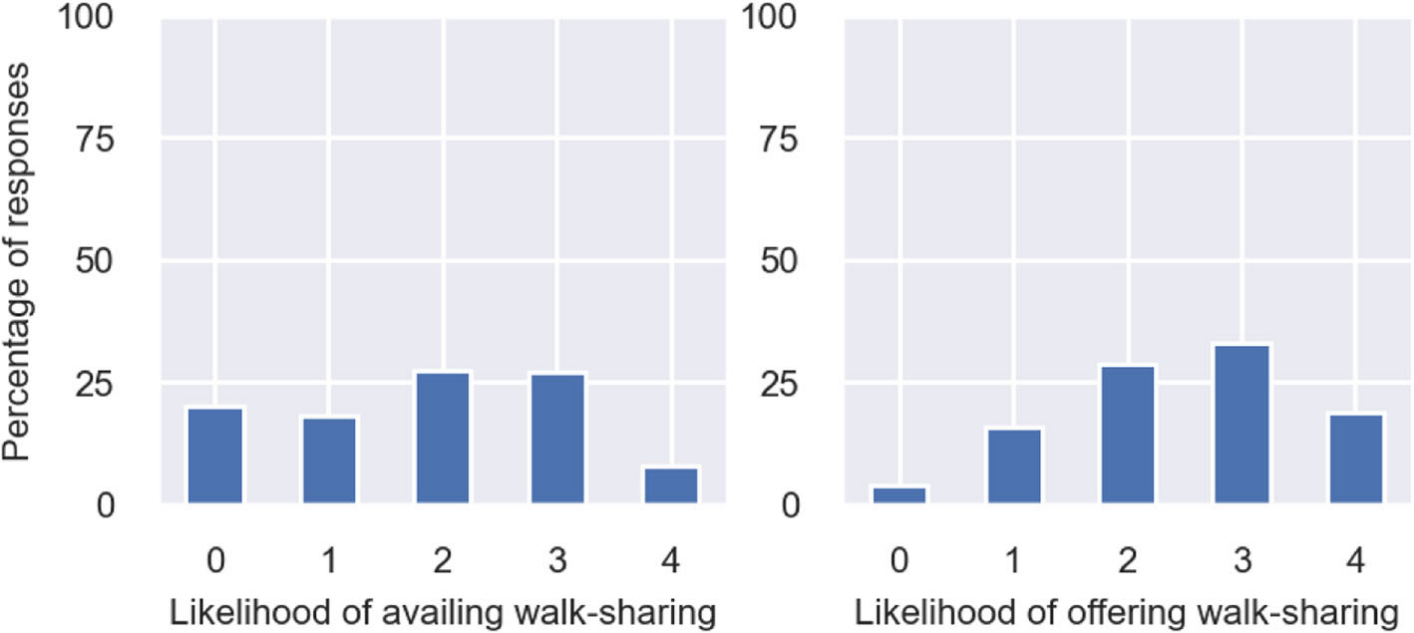
Distribution of responses on likelihood of availing and offering walk-sharing (0 = Highly unlikely, 4 = Highly likely)

We wanted to understand to what extent are people interested to avail walk-sharing. Hence, we asked them to state on a Likert Scale of 0 to 4, how likely they are to avail walk-sharing, 0 being *Highly unlikely* and 4 being *Highly likely*. It can be observed from Fig. 8 that responses of people is well-distributed across the Likert scale with most people stating they are *Neutral* to *Likely* to avail walk-sharing. In contrast, when we asked people whether they are willing to offer walk-sharing, given that other people might exist who may need to avail it, people responded more positively, with significant improvement in the share of people who stated *Highly likely*. This indicates that a portion of people feel that they are relatively confident while walking alone, but at the same time, are willing to participate in walk-sharing when others need it.

**Fig. 9:**
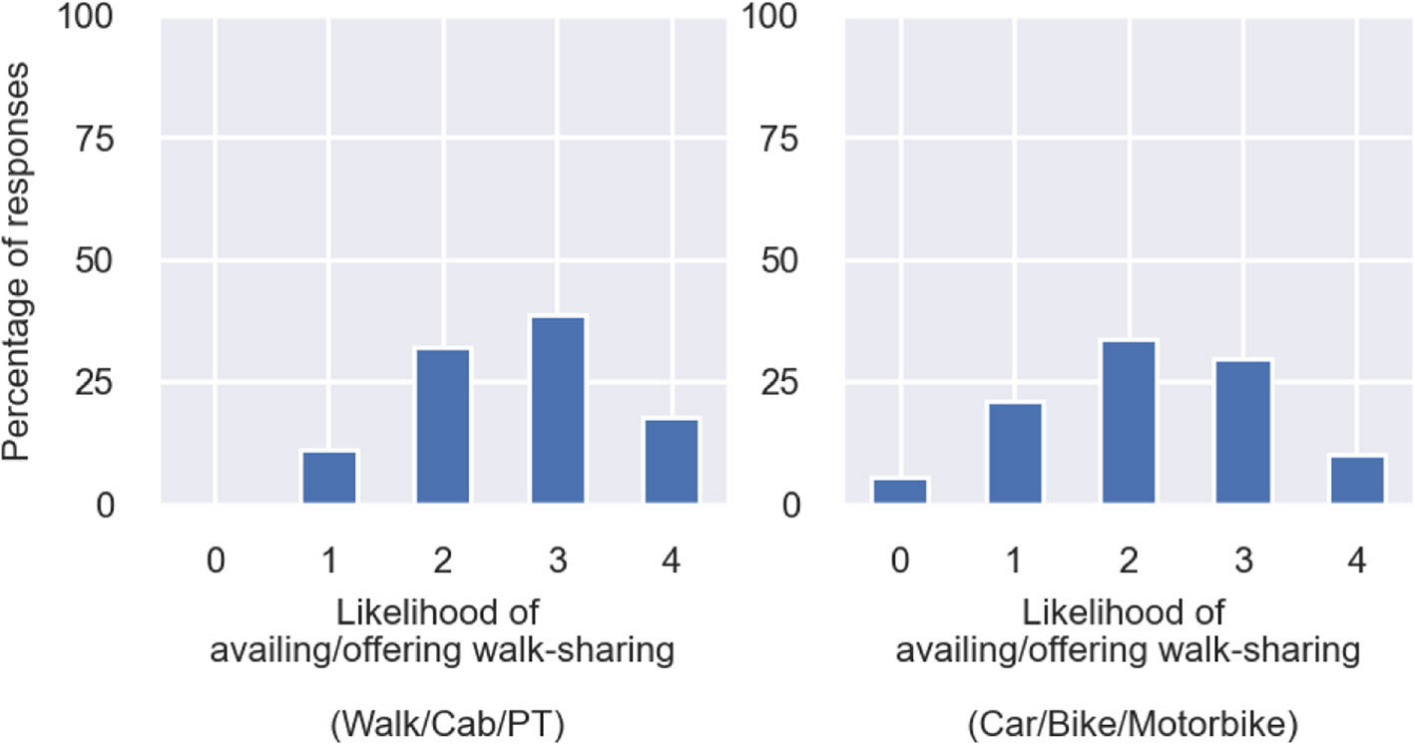
Distribution of responses on the Likert scale by existing mode of travel (0 = Highly unlikely, 4 = Highly likely)

Consequently, we also wanted to understand whether demographics drive the likelihood of taking up walk-sharing. We used independent samples t-test to investigate whether the mean likelihood score, calculated using the mean of the stated responses against likelihood of taking up walk-sharing, was significantly different across any two demographic groups. We conducted the tests using governing demographics *age, gender, educational qualification and existing mode of travel*. We found that the *existing mode of travel* is the only factor that is associated with the stated response against likelihood of availing or offering walk-sharing. We divided the responses into two independent samples, one group of people who used private modes of transport such as a car or motorbike or bike to travel to their most commonly visited supermarket, and the other group who availed either public transport or cab or simply walked to their nearest supermarket. We found that the mean likelihood score (on the Likert scale of 0 to 4) to avail walk-sharing was significantly higher (at 99% confidence interval) for the respondents whose existing mode of travel is either public transport, a cab or simply walking. This means that people who do not usually use their private vehicle, are more likely to avail walk-sharing. On the other hand, people who travel using their private vehicles, are less willing to switch to walk-sharing as travel mode. This can be supported by findings from existing studies which suggest that people who drive more often have lower distance thresholds for walk trips, and consequently walk less (Ralph et al. 2020). The distribution of responses for these two groups are shown in Fig. 9.

## Model calibration for university scenario

This section talks about how we have calibrated our existing agent-based model that was developed and used in our previous study (Bhowmick et al. 2021) based on the evidence obtained from the results of the survey on stated preferences of people related to walk-sharing. In the following subsections, we discuss briefly about the campus Wi-Fi dataset used for the simulations, the walk-sharing model, the revised parameter thresholds of the agent-based walk-sharing model and the modification of the matching algorithm inside the model.

### Suitability of walk-sharing for university campuses

While a university campus may seem apparently safer than the streets surrounding it, this perception could be argued against on multiple fronts. First, many public university campuses are not gated, so access and egress to the outdoor areas of the campus is seamless. Second, even if the campus has stricter access controls, students or staff walking to public transport stops must traverse a significant portion of their trip outside the campus. Third, there have been safety issues raised consistently, even inside university campuses. Hence, actual and perceived risk does not always vary significantly across the geographical boundaries of the campus. Walk-sharing seems suitable for such a scenario. University campuses and surrounding areas are more walkable in general. They usually cater to numerous pedestrian trips, because walking is the first leg of most journeys. So sufficient pedestrian demand and relative spatial proximity between people inside a university campus, clubbed with existing safety challenges, make university campus a favourable location (or scenario) where walk-sharing could be both practically viable and effective in terms of perceived and actual risk reduction.

### Campus Wi-Fi data

**Fig. 10:**
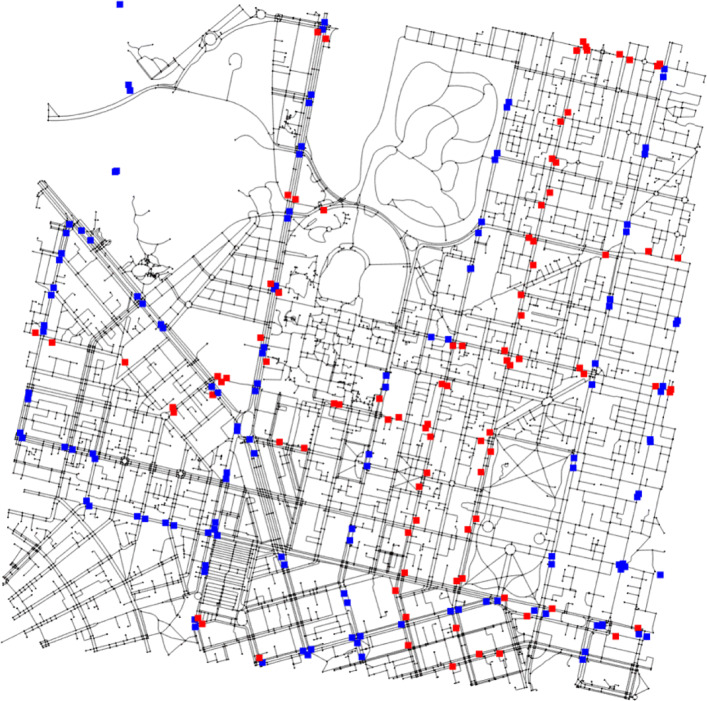
Pedestrian network in and around The University of Melbourne, Parkville campus; blue squares refer to tram stops, red squares refer to bus stops

The University of Melbourne collects data about devices accessing their on-campus Wi-Fi network for the purpose of space management. In the event of a device accessing (probing) the university’s Wi-Fi network, the details of this action are recorded along with relevant data. This information is securely retained in the university’s database. We had obtained a completely anonymised dataset from the university containing the last probing event of every device every day. These include devices used by staff and students inside the Parkville campus of the university for every day of the year 2019. The dataset contains 12.14 million records for the calendar year of 2019. This amounts to approximately 34k *last seen* records per day with 208,667 unique devices probing throughout 2019. The spatial granularity was at the building level while the temporal granularity was at the minute level. Necessary preprocessing steps were conducted to deduce the number of people from the number of devices, so that these daily *last seen* records can act as an appropriate proxy data for people exiting the campus from different buildings at different times of the day. Due to privacy concerns, access to the personal level data was not granted by the dataset providers, and hence identifying multiple records resulting from a single person was challenging by ourselves. As per our request, a preliminary analysis was conducted by the dataset providers at the university to estimate the average device-to-person ratio for the entire dataset, given their access to privacy-sensitive personal data as well. Static devices were first filtered out by the dataset providers, as well as probing events to outdoor Wi-Fi receivers. Since people usually carry more than one device with themselves that is connected to the Wi-Fi network (e. g. a smartphone and a laptop), some analysis was necessary to estimate the number of people from the number of probing events. They arrived at an average value of 1.8 devices per person. Hence, 45% of the records for any given hour of a given day were removed randomly (based on the obtained ratio of 1.8 devices per person) before conducting the simulation. For our simulations, we proceed with our previous selction of three days viz. 2^*nd*^ February, 21^*st*^ November and 11^*th*^ April. The choices are such as they correspond to the 5^*th*^*percentile*,50^*th*^*percentile* and 95^*th*^*percentile* of daily exit record counts, respectively. Data on the pedestrian network surrounding the university campus was obtained using OpenStreetMap. The network has been illustrated in Fig. 10.

### Modified parameter thresholds

**Fig. 11:**
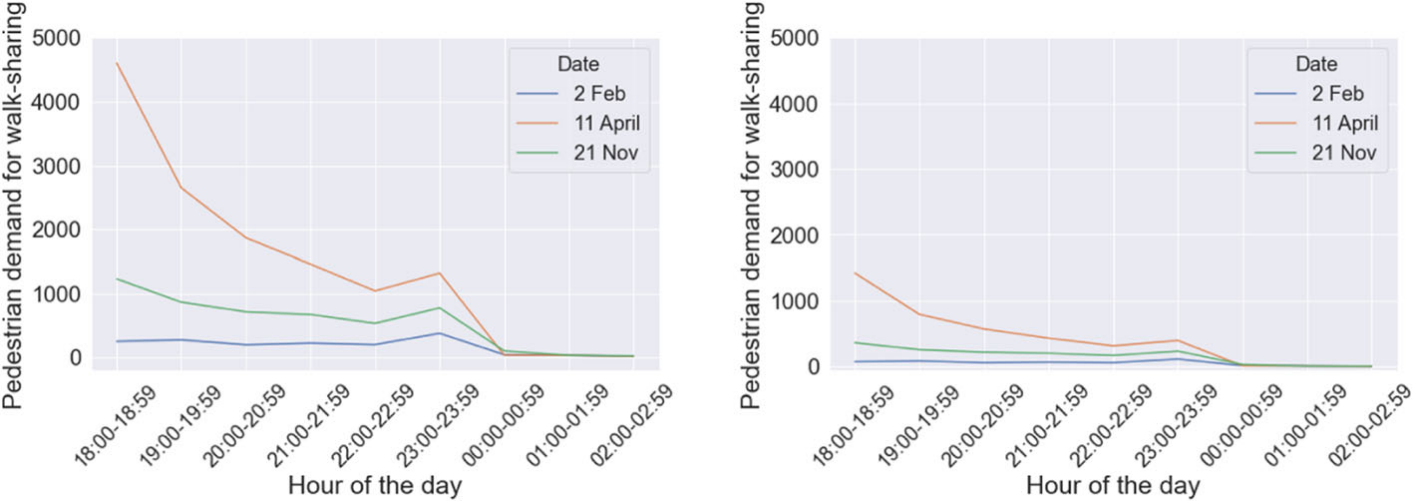
Number of agents willing to avail walk-sharing, before introducing IAR (left) and after IAR (right)

In our previous study, we had assumed that all the agents in the study are willing to participate in the walk-sharing scheme, since we had limited information on how people may perceive walk-sharing. We understand that there will be a proportion of people who would not be willing to participate in walk-sharing even if it satisfies their space-time constraints. Hence, we have defined the *Initial Acceptance Rate (IAR)* for our dataset, which captures (sufficiently) the whole population on campus. IAR is the proportion of agents from a population who are willing to participate in walk-sharing, provided it satisfies their space-time and social constraints. The rest of the agents will not avail it anyway. Based on our analysis that the likelihood of availing or offering walk-sharing is strongly associated with the existing travel mode, we have divided the agents into two separate classes. From the responses received through our survey, 34 out of 53 full-time students (64%) and 77 out of 142 part-time and full-time workers (54%) said that they either walk or avail public transport for commute. As per the 2019 Annual Report of The University of Melbourne, 9380 (14%) staff were employed with the student load standing at 54714 (86%). We multiplied the survey percentages with the university’s numbers to obtain the share of agents who walk to their destination or avail public transport. That estimate comes to approximately 63% (64% of 86 added with 54% of 14). Given that the university campus holds ample walkable spaces and transit services around it, the number of people walking will be higher than the general population, as represented by the survey. Therefore, we scale up the share from 63% to 70%. In summary, we have assumed that 70% walk to their nearest public transport stop while 30% of the agents use their personal vehicle, either a car or a bike. Given the distribution of the responses appear to be normally distributed, we calculated the mean likelihood score for the two classes. For the first class of agents who avail public transport, the calculated mean likelihood score is 2.63 with a standard deviation of 0.9. For the second class of agents who use their personal vehicles, the calculated mean likelihood score is 2.18 with a standard deviation of 1.05. Based on these statistics, we assigned a likelihood score to each agent. Then, we calculated IAR by counting the proportion of agents across both classes whose assigned likelihood score was 3.0 or more. This means we only consider agents who are either *Likely* or *Highly likely* (based on Likert scale) to participate in walk-sharing. Consequently, pedestrian demand for walk-sharing has reduced to roughly 30% of the demand that was used in our previous model. This can be observed from Fig. 11. We discard the rest of the agents from the simulation.

For the maximum waiting time threshold, we had observed that the distributions were significantly contrasting when split using *place of birth*. We saw that people born in Australia were temporally more flexible as compared to the people born outside Australia. However, we were unable to support this finding with established evidence, given its non-trivial nature and the socio-demographic diversity within the migrant population in Australia. We reviewed relevant literature, mostly involving ridesharing, but were unable to find any similar findings. Instead of speculation, we have attempted to replicate the maximum waiting time distribution obtained from the survey, by using a log-normal distribution up to 15 minutes. We have used the observed mean (6 minutes) and standard deviation (4 minutes) from the survey. Since 30% of respondents have stated their willingness to wait more than 15 minutes, we have defined a separate class of agents whose maximum waiting time varies uniformly within the range of 16-20 minutes.

**Fig. 12:**
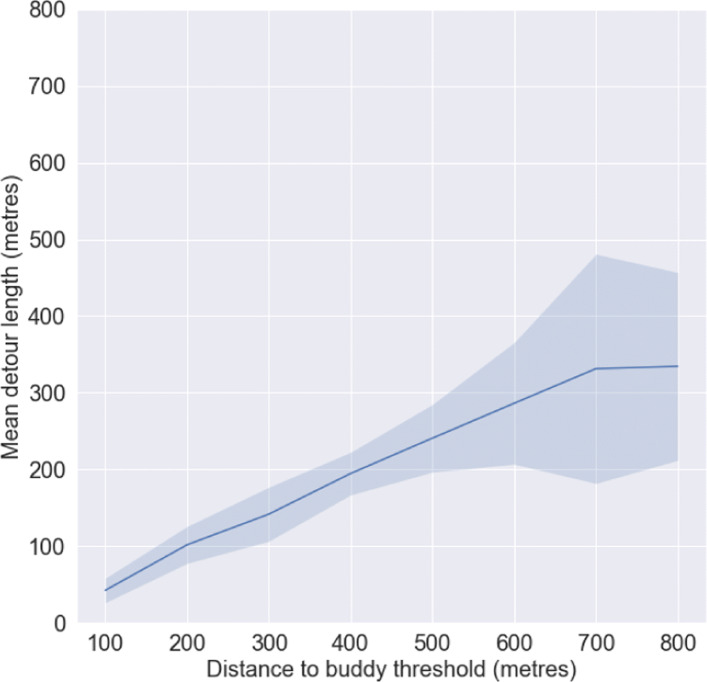
Detour length vs Distance tobuddy threshold for 21^*st*^ November; mean calculated over 6 PM in the evening to 3 AM in the morning, 2 standard deviations shown

We had calculated a mean detour time threshold preference of 6 minutes from the set of responses which is equivalent to 432 m detour length, assuming a mean walking speed of 1.2 m/s (VicRoads 2019). The best proxy variable for detour length in our model is *distance to buddy threshold*. Using data from 21^*st*^ November (50^*th*^ percentile demand day), we simulated the variation in mean detour length (mean calculated over 6 PM in the evening to 3 AM in the morning) and distance to buddy threshold. Using the results shown in Fig. 12, we observed a peak detour length of 331 m at a distance to buddy threshold of 700 m. 331 m is equivalent to a detour time of roughly 5 minutes which is close to 6 minutes, the approximate mean value obtained from the survey. Hence, we set the distance to buddy threshold of our model at 700 m.

As per the 2019 Annual Report of The University of Melbourne, the percentage of female student enrolments was approximately 57% while the share of women staff was also 57%. Gender classification was binary (Male and Female), given we had only two respondents out of 234 who identified themselves as non-binary. Given the representation of the non-binary gender was not substantial, we have limited our gender classification to a binary one (either male or female). Hence we have assigned a probability of an agent being a female one as 0.57 and that of being a male as 0.43. The parameter thresholds set for the calibrated model are summarised as follows:

- Existing modes of travel for agents (influencing IAR):
  - Walking to PT stop = 70%
  - Car and bike = 30%
- Maximum waiting time
  - Normal agents = ln (lognormal (6 *min*,4 *min*))
  - Flexible agents = uniform (15 *min*−20 *min*)
- Distance to buddy threshold = 700 m
- Distance to destination threshold = 700 m
- Gender
  - Male = 43%
  - Female = 57%
- Same gender preference probability
  - Male = 0.1 (10% of male agents prefer only male agents)
  - Female = 0.5 (50% of female agents prefer only female agents)

The comparison between the parameter thresholds of the initial model developed in the previous study and the calibrated model is illustrated in Table 2.

**Table 2:** Comparison of parameter thresholds between the initial model developed in the previous study and the calibrated model

Parameter	Value in initial model	Value in calibrated model
Maximum waiting time	Uniformly varied between 5-10 min	Normal agents
		= ln (lognormal (6 min, 4 min))
		Flexible agents
		= uniform (15 min - 20 min)
Distance to buddy threshold	200 m	700 m
Distance to destination threshold	700 m	700 m
Existing travel modes	NA	Walking to PT stop = 70%
		Car and bike = 30%
Gender	NA	Male agents = 43%
		Female agents = 57%
Same gender preference probability	NA	Male agents = 0.1
		Female agents = 0.5

### Modified matching algorithm

The matching algorithm has been modified to accommodate for the gender preference of the agents. In the base model, any agent could be matched with any other agent given they satisfied some spatio-temporal constraints, such as distance to buddy threshold or maximum waiting time. In this instance, there is presence of sufficient evidence regarding social preference of people, with almost half of the female respondents stating that they prefer a walking companion of the same gender. This same-gender preference is substantially lower, only 10% in the case of male respondents. Gender preference was randomly assigned to all agents, with all female agents having a 50% chance of having same-gender preference, and male agents having a 10% chance. In the matching step, agents having same-gender preference can only be assigned to another agent of the same gender. Hence, while constructing the distance (impedance) matrix, it was checked whether the two agents in question are of the same gender. If not, a check is made to see whether either one of them have a same-gender only preference. If that is the case, then to nullify the chances of a match, we replace the corresponding distance value in the matrix with a large positive value. We have stated this modification between Line ?? - Line ?? in Algorithm 1. The rest of the algorithm remains the same as mentioned in (Bhowmick et al. 2021).


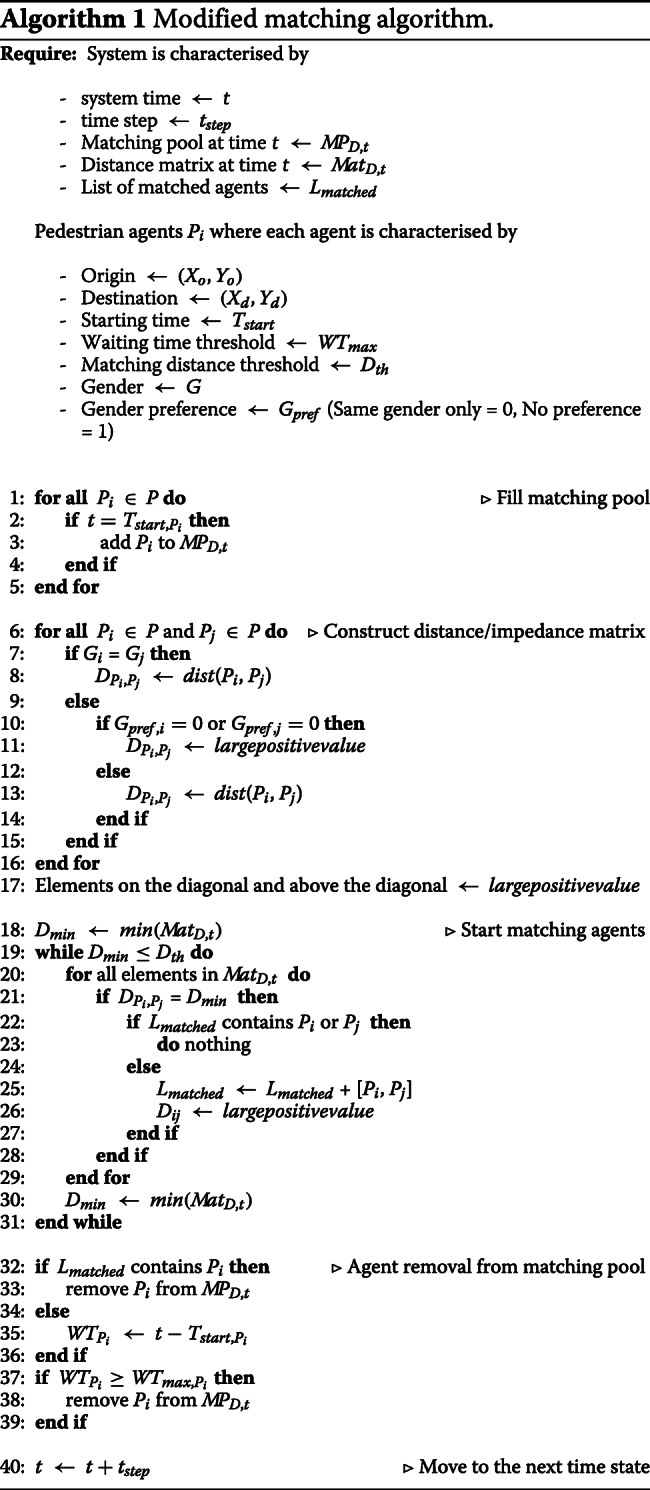


## Results

Integrating the Wi-Fi data into the calibrated agent-based walk-sharing model, ensuring the parameter thresholds had been adjusted to take account of the survey results, we ran the simulations for each scenario. We mitigated the stochasticity involved in temporal and social preference assignment to the agents by repetitive simulation runs, and consequently taking the average. We have provided a comparison for each of the performance metrics, viz. waiting time, walk-alone distance, detour distance, matching rate and finally, safety index.

### Waiting time

**Fig. 13:**
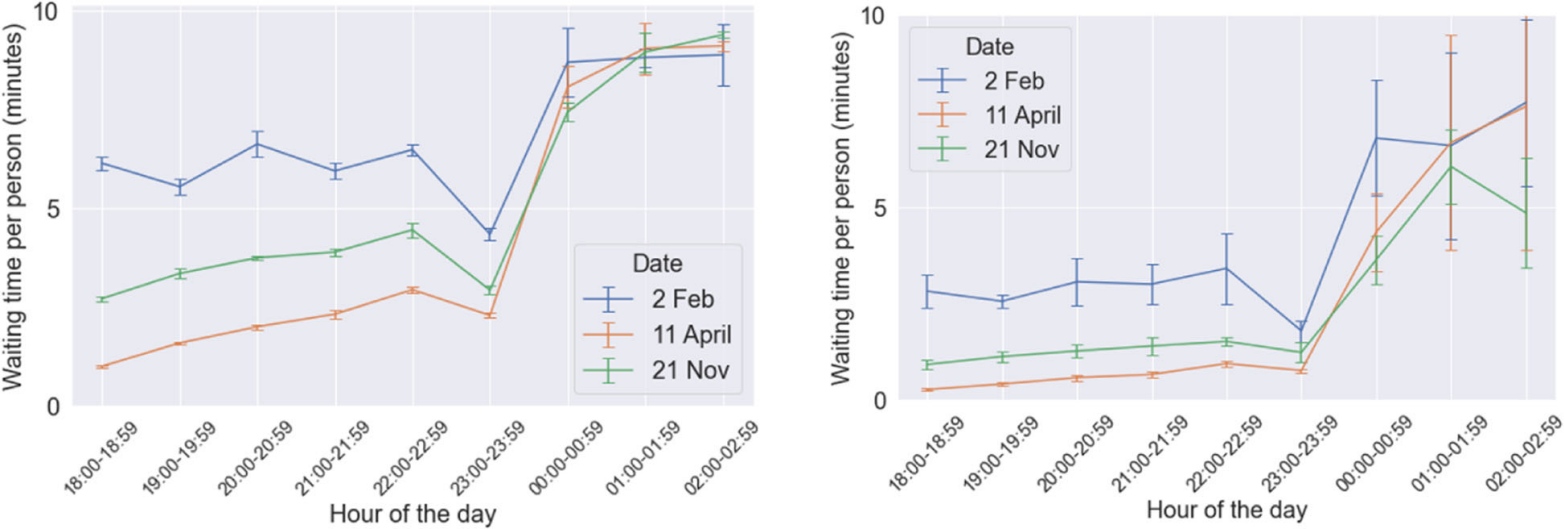
Results of mean waiting time per person (one standard deviation error bars shows) from the initial model (left) and the calibrated model (right)

It can be observed in Fig. 13 that mean waiting time per person has reduced considerably in the results obtained from the calibrated model, as compared to the results from the initial model, viz. the model used in (Bhowmick et al. 2021). The mean is taken over all agents present in the system, irrespective of the fact whether they were matched or not. This can be primarily attributed to the fact that *distance to buddy threshold* has been increased from 200 m to 700 m given that respondents have stated that they are more flexible in terms of detour distance. We had observed in our previous study that waiting time per person increased with lower pedestrian demand but reduced with higher values of distance to buddy threshold. While demand has been substantially low, it was still enough to not increase waiting time significantly, while the fact that *distance to buddy threshold* was increased from 200 m to 700 m had a greater weight and eventually resulted in significantly lower waiting times. Till midnight, waiting time remains well below 5 minutes, and after that it ranges from 5 minutes to 10 minutes. These are acceptable values, given that the stated responses from the survey exhibited a mean of 10 minutes as the maximum preferred waiting time (see Fig. 3).

### Walk-alone distance and detour length

**Fig. 14:**
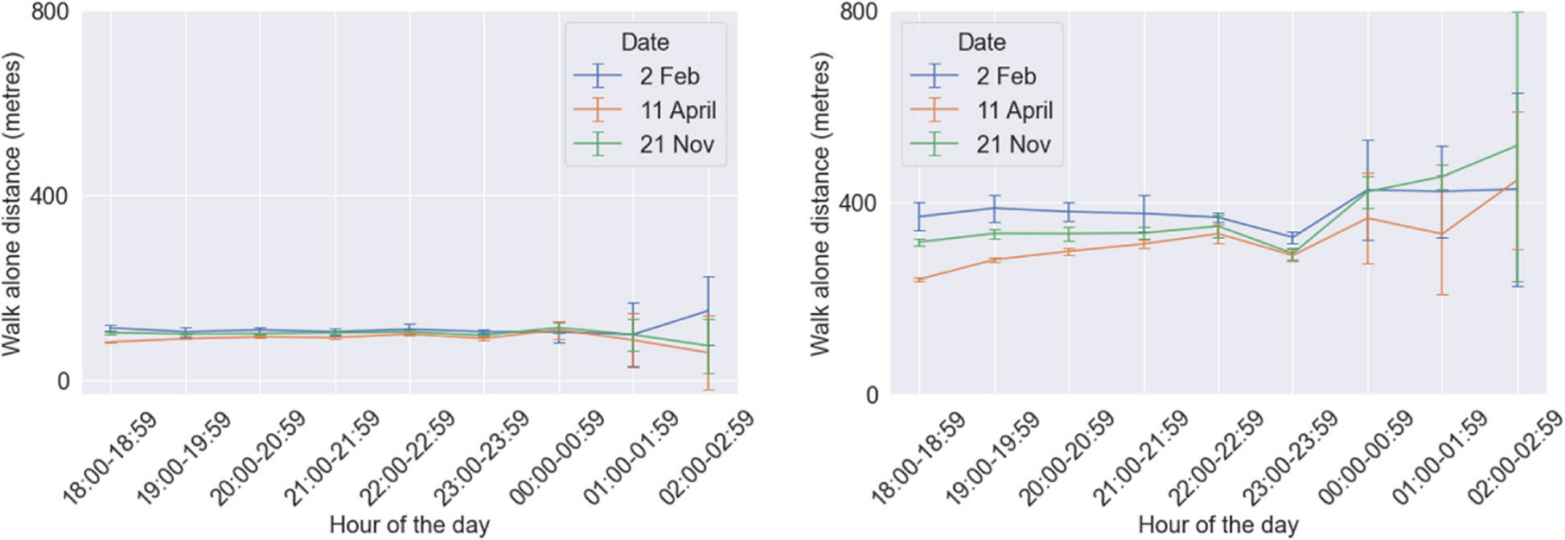
Results of mean walk-alone distance per person (one standard deviation error bars shows) from the initial model (left) and the calibrated model (right)

**Fig. 15:**
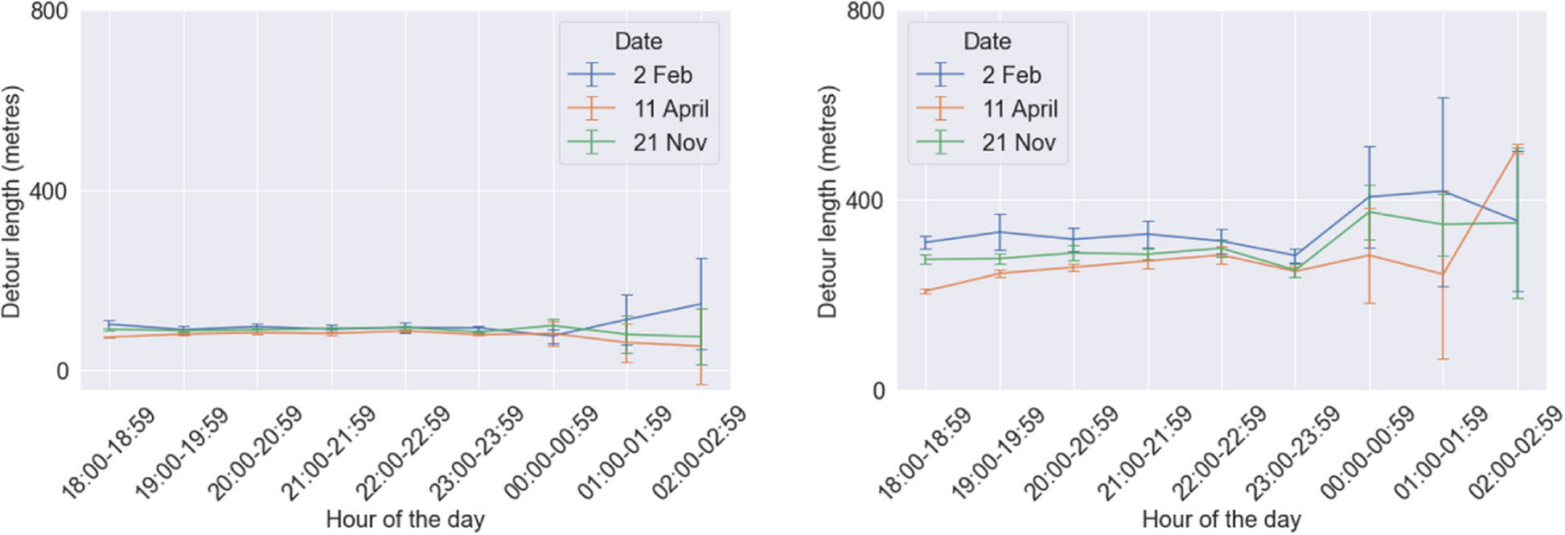
Results of mean detour length per person (one standard deviation error bars shows) from the initial model (left) and the calibrated model (right)

The calibrated model exhibits significantly higher values of walk-alone distance (Fig. 14) and detour length (Fig. 15) when compared to our initial model. This occurs due to two reasons. First, *distance to buddy threshold* has been increased from 200 m to 700 m in the calibrated model as per the responses received from our survey. So people are more flexible to be matched with potential companions who are farther away. It was observed in the theoretical developments of our previous study that distance to buddy threshold has a substantial impact on both detour length and walk-alone distance. Hence, it is the major reason behind the significant rise in these two performance metrics. Second, the incorporation of same-gender preference in the agents has resulted in many nearby matches being invalid (cases where two agents belonged to different genders), and thus forced into matches possibly farther away. Yet, both walk-alone distance and detour length remain in the range of roughly 400 m corresponding to less than 6 minutes of walk-alone time or detour time. We had observed from the responses in the survey that the mean detour time was roughly a little over 6 minutes (see Fig. 3). Hence, while both walk-alone distance and detour length have increased thereby reducing performance, still they remain within the acceptable thresholds stated by the community.

### Matching rate

**Fig. 16:**
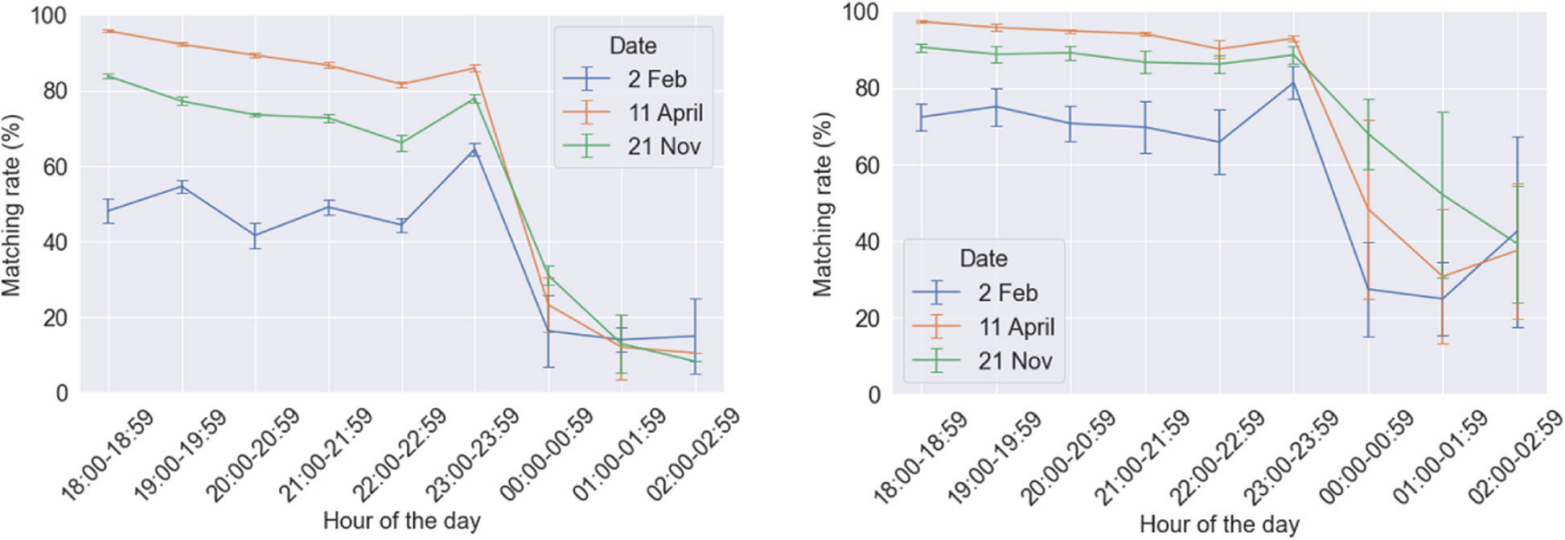
Results of system matching rate (one standard deviation error bars shows) from the initial model (left) and the calibrated model (right)

Matching rates have improved in the calibrated model as can be observed from Fig. 16. This is more significant for 2^*nd*^ February which corresponds to the 5^*th*^ percentile demand day. For this day, in the initial model, matching rate varied between 40-60% before midnight, whereas in the calibrated model, matching rate stays well above 60%. In our previous study we had observed that matching rate decreases with reduction in demand. Also, matching rate reduces due to incorporation of social preferences, which makes the number of suitable matches fewer. But, the significant increase in matching rate is due to an increase in distance to buddy threshold. This is in line with our observations from our previous study, where an increase in distance to buddy threshold lead to sharp improvements in matching rate, even in low-demand scenarios.

### Safety index

**Fig. 17:**
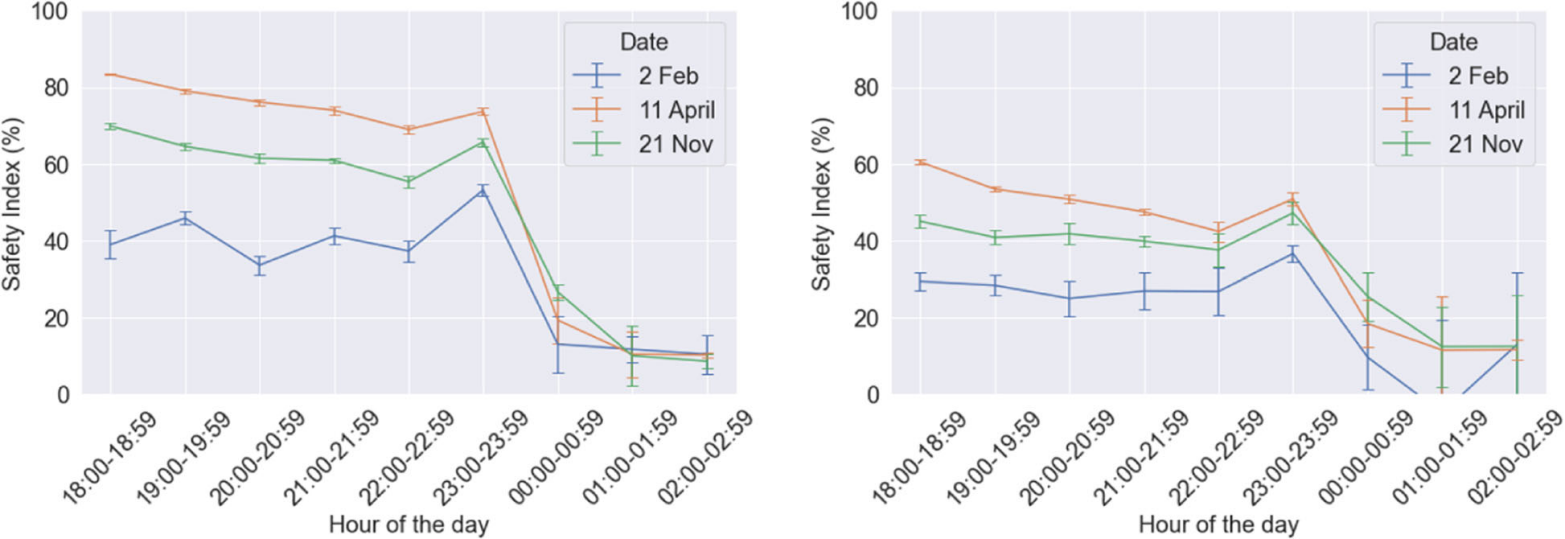
Results of safety index (one standard deviation error bars shows) from the initial model (left) and the calibrated model (right)

In our previous study, we had defined *safety index* as the “*mean walk-alone distance saved per capita expressed as a percentage of the distance walked by a person in the absence of walk-sharing*". Safety index acts as a proxy for objectively measuring the extent of safety improvement done by walk-sharing. It can be observed in Fig. 17 that the safety index values before midnight has declined slightly, when compared to results from our initial model. This is a resultant of increased distance walked alone by the agents, due to greater spatio-temporal flexibility. Considering the 21^*st*^ November which corresponds to the 50^*th*^ percentile pedestrian demand, the safety index varies between 40-50% before midnight, a reduction from 60-70%. This means that for half of the days, walk-sharing can contribute towards 50% improvement of pedestrian safety perception and thus reduced fear of crime while walking.

## Discussion

The results obtained from the calibrated model, where the parameter selection was more informed, shows that walk-sharing has the ability to make a significant improvement in pedestrian safety perception levels in a real-world scenario, while exhibiting acceptable values of other performance metrics. This finding is in line with our claims from our earlier study (Bhowmick et al. 2021) where we showed a possible maximum value of walk-sharing’s capacity to improve pedestrian safety. We had expected, that after calibration, the performance of walk-sharing will reduce somewhat. While the performance has reduced in terms of walk-alone distance, detour length and safety index, we have also seen significant improvements in terms of waiting time and matching rate. Both waiting time and detour time remained within the preferred thresholds of the survey respondents, which could improve the appeal of walk-sharing. It must be noted that we had seen in the theoretical findings of our previous study that the performance of walk-sharing diminished significantly in the face of lower pedestrian demand levels. With the inception of Initial Acceptance Rate, the pedestrian demand had reduced to 30% of the magnitude of our previous model. Apart from that, the incorporation of social preference, viz. the same-gender preference criteria, rendered many matches invalid, many of which were perfectly acceptable in our previous model. While these have driven down the performance of walk-sharing on one hand, what boosted its performance on the other hand was the increased spatio-temporal flexibility of people, which we had underestimated previously, in terms of distance to buddy threshold, and greater distribution of waiting time threshold, now ranging up to 20 minutes. Overall, we see that, in spite of the matching complexities introduced, walk-sharing is able to establish its practical viability to improve safety perception and safety of pedestrians.

There are certain aspects of the study which could be viewed as limitations, and therefore, can be improved upon in future. Firstly, the simulations were conducted with mobility data from the university campus. Usually, university campuses are more walkable, and involve numerous pedestrian journeys. This makes it more conducive to walk-sharing as performance of walk-sharing varies significantly with pedestrian demand and spatial proximity of pedestrians. Accordingly, walk-sharing was found viable in the university scenario. But it has not been tested in other, possibly more challenging scenarios. The base model is transferable and can be calibrated as per the requirements of other critical scenarios, given the availability of appropriate mobility data. Also, in our modelling, we have assumed that the stated spatio-temporal and social preferences of a person under critical circumstances, is independent of where this circumstance occurs. This means, preferences are assumed to be transferable from scenario to scenario. As per this assumption, the takeaways from the stated preference survey were transferable to the university scenario, and therefore fed unmodified into the model. Second, to extract community perception on walk-sharing, we conducted a web-based questionnaire survey. Given walk-sharing is at its conceptual stage, we had to employ stated-preference (SP) methods, defining new variables, costs and performance metrics. While SP methods are prone to biased responses, deducing the amount of such bias is challenging, costly and time-consuming and do not fall within the scope of this study. Also, the survey was conducted in an online platform due to COVID-19 restrictions, and online survey platforms have their own shortcomings. Third, the survey was limited to only Australian respondents. This was primarily due to limited funds that could only accommodate a certain number of people. Within that limited set of respondents, we wanted to avoid challenges corresponding to socio-economic and cultural diversities governing the preferences related to walk-sharing. Finally, there were some trends in the survey that were difficult to explain. For example, we found it challenging to understand how a person’s place of birth correlates with their spatio-temporal flexibility. Hence, we did not incorporate this finding into our model. While this may not influence the performance of walk-sharing significantly in the university context, such information could help us better understand efficient implementation of walk-sharing.

## Conclusions

With increased fear of crime among pedestrians, walk-sharing provides an alternative intervention that promises significant improvements in the urban walking experience. Walk-sharing aims to reduce fear of crime and thereby enhance pedestrian safety and safety perception. Walk-sharing has its distinct advantages over its traditional counterparts, given that it is more inexpensive, less data-dependent, holistic and scalable. While our previous study had outlined the theoretical framework of walk-sharing and proved its technical viability, it fell short in terms of not considering public acceptance and feedback about the same. To plug this research gap, we conducted a web-based survey to understand public perception on walk-sharing. We have presented a summary of the responses and the results derived from analysing the same. Given that public perception on walk-sharing was never studied before, these results were interesting. Nevertheless, the objective of our study was not limited to the mere presentation of survey results. We had planned to incorporate these findings in to our walk-sharing model and calibrate it to understand whether walk-sharing can be practically viable in a real-world scenario. Our calibrated walk-sharing model delivered promising results even under significantly low pedestrian demand levels and more complex matching circumstances. Walk-sharing alone is still able to improve pedestrian safety in the range of 20-60% (more than 40% for half of the days) in the university scenario. It achieves these figures keeping other performance metrics such as matching rate, waiting time, walk-alone distance and detour length under acceptable thresholds. In an age of ubiquitous computing, IoT, efficient location-based services and smartphones, walk-sharing could be the new-age solution that brings people back to the sidewalks, promote walking as not only a healthier mobility choice but a safe one as well, and consequently progress towards more sustainable urban living, by reducing short-distance motorised traffic.

Future work will involve improvements to the walk-sharing algorithm by making it more sophisticated, better optimisation of the matching algorithm, suggesting landmarks as possible meeting points, understanding the sensitivity of walk-sharing to other origin-destination flow structures, and testing the viability of walk-sharing in other urban scenarios. Validation of survey findings could also be executed in future studies. Other possible improvements include surveying people from different countries and conducting some face-to-face interviews to get a deeper understanding of public perception.

## Data Availability

The Wi-Fi dataset used in this study was obtained from The University of Melbourne. The authors do not have the rights to share this dataset with anyone. The relevant codes with sample encrypted data files are available in this Github repository.
